# Effects of prebiotics in combination with probiotics on intestinal hydrolase activity, microbial population and immunological biomarkers in SD rats fed an AIN-93G diet

**DOI:** 10.1186/s42826-022-00132-5

**Published:** 2022-07-18

**Authors:** Min-Jeong Kim, Dong-Gyeong Jeon, Yong Lim, Insurk Jang

**Affiliations:** 1 Division of Animal Bioscience and Integrated Biotechnology, Gyeongsang National University, 33 Dongjin-Ro, Jinju, 52725 Gyeongnam Korea; 2 Department of Clinical Laboratory Science, Dong-Eui University, Busan, 47340 Korea

**Keywords:** Prebiotics, Probiotics, GI tract, Microbiota, Immunity

## Abstract

**Background:**

Gastrointestinal microbiota, which comprises hundreds of different types of microbes, biologically plays crucial roles in the host’s health. Probiotics (PRO) did not always have a positive benefit on the host, depending on strains of microbes and the physiochemical properties of prebiotics (PRE), indicating that the properties of PRE in combination with PRO might have different effects on the gut ecology. The aim of this study was to assess the effects of insoluble or soluble PRE with PRO on intestinal digestive hydrolase, the fecal microbes, and immunological biomarkers in SD rats fed an AIN-93G diet.

**Results:**

Forty, 8-week-old SD rats were randomly assigned to 4 groups with 10 replicates in each; cellulose (CELL), cellulose + probiotics (CELPRO), oatmeal (OATS), and oatmeal + probiotics (OATPRO) groups. After 4-week feeding trial, rats were treated with saline or lipopolysaccharide (LPS, 1 mg/kg) to examine the alleviating effects of PRO and PRE on immunological responses. There was a significant (*p* < 0.05) decrease in feed intake of rats fed the oatmeal supplemented diet without affecting growth performance. Blood triglyceride was significantly (*p* < 0.05) decreased in rats fed the oatmeal diet, and aspartate aminotransferase (AST) was significantly (*p* < 0.05) decreased in rats fed the PRO supplemented diet. Intestinal maltase, sucrose, and lactase activities were significantly (*p* < 0.05) higher in rats fed PRO compared with rats not fed PRO. Rats fed the oatmeal showed a significant (*p* < 0.01) increase in the fecal colony forming units (CFU) of *Lactobacillus plantarum, Bacillus subtilis, and Saccharomyces cerevisiae* compared with those fed cellulose. LPS-treated rats fed PRO showed a significant (*p* < 0.05) increase in blood secretory immunoglobulin A (*s*IgA) compared with those not fed PRO. The LPS-treated rats fed PRO resulted in decreased (*p* < 0.05) blood IL-6 compared with those not fed PRO, indicating that a dietary PRO alleviated inflammatory response in LPS-treated rats.

**Conclusions:**

Dietary oatmeal increased fecal microbes, and PRO supplement resulted in increased intestinal hydrolase and immune functions of the host, demonstrating that soluble PRE with supplemented with PRO could be a more bioactive combination of synbiotics in SD rats.

## Background

Gastrointestinal (GI) microbiota consists of hundreds of different types of microbes and biologically plays multiple roles in intestinal milieu of animals [1]. Numerous studies demonstrated that the modulation of gut microbiota remarkably affected the host health [2]. At present, the symbiotic relationship between the host and gut microbiota is currently an active investigation area due to its application to improve gut diseases such as diarrhea, colitis, and inflammation in the host [1, 3, 4]. Probiotics (PRO) is a direct-fed live microbial supplement, which beneficially improves the microbial ecosystem in the GI tract of animals [4]. Many studies have reported that commonly using PRO containing *Lactobacilli (L.)* and *Bacillus (B.) subtilis* strains improves intestinal functions and immunity in the host [5, 6]. *Saccharomyces (S.) cerevisiae*, one of the most crucial PRO has been also reported for enhancing specific and non-specific cellular immunity in various species of animals [7, 8]. The proposed action modes of PRO establish beneficial gut microbiota and activate immune system via the suppression of intestinal pathogens, secreting intestinal immunoglobulin A, and decreasing inflammatory responses in the GI tract [9]. However, the beneficial effects of PRO on the host’s health varied greatly according to genus and strain specific manners of microbes [1, 10]. Thus, the combination of beneficial microbial species including *Lactobacilli, B. subtilis, S. cerevisiae*, etc. has been extensively studied to maximize the host’s health.

Prebiotics (PRE), fiber components including β-glucan, oligosaccharide, inulin, cellulose, etc., are well recognized to have beneficial effects on the host by altering microbiota composition and fermented metabolites in the GI tract as reported by many studies [11, 12]. As a result of this promising result, the combination of PRO and PRE known as synbiotics has drawn tremendous attention. Synbiotics are designed to formulate both PRO and PRE to overcome some possible problems in the settlement of PRO in the GI tract. Considering an appropriate combination of these supplements, synbiotics may ensure a synergistic effect on the host health compared with PRO or PRE alone, since PRE can be used as the substrates for the growth of PRO in the GI tract. It has been reported that synbiotics stimulate the growth of beneficial microbes and inhibit pro-inflammatory cytokine including the tumor necrosis factor-α (TNF-α) in the GI tract [13].

As with the PRO studies, dietary synbiotics did not always have a positive benefit on the host health, depending on the physiochemical properties of PRE. The properties of PRE in combination with PRO might have different effects on the gut ecology. Dietary PRE has been divided into soluble and insoluble fibers. Soluble fibers such as β-glucan are easily fermented by gut microbes in the lower GI tract. Insoluble dietary fibers including cellulose do not easily form gels due to its water insolubility and hardly ferment in the GI tract [14]. The different properties of PRE may lead to a different composition of gut microbiota and fermented metabolites [15], suggesting that the effect of synbiotics on intestinal milieu varies greatly depending on the type of PRE present in PRO. This study was conducted using AIN-purified diet in rats to control dietary PRE components in feed ingredients.

Therefore, the aim of the study was to assess the effects of insoluble (cellulose) or soluble (oatmeal) PRE in combination with PRO as synbiotics on intestinal functions including digestive hydrolase, the fecal microbes, and immunological biomarkers in rats fed an AIN-93G diet.

## Results

### Growth performance and organ weights

Body weight, gain and FCR in response to dietary PRE and PRO were monitored in rats during the 4-week experimental period (Table 1). Body weight, gain, and FCR on a cumulative basis were not affected by the PRE or PRO supplement. However, there was a significant effect (*p* < 0.01) of PRE type (cellulose vs. oatmeal) on feed intake. Rats fed the diet containing oatmeal as PRE type showed significantly (*p* < 0.01) lower feed intake compared with those fed the diet supplemented with cellulose. In addition, gain during the 4-week feeding trial tended to be lower (*p* < 0.09) in rats fed an oatmeal supplemented diet. When the effect of dietary PRE and PRO on organ weights of rats was measured, the weights of the liver, spleen and small intestinal mucosa were unaffected by PRE and PRO (Table 2).

**Table 1:** Effect of prebiotics and probiotics on growth performance, feed intake and feed conversion ratio (FCR)

Item	Treatment group*				Significance (*p* value)		
	CELL	CELPRO	OATS	OATPRO	PRE	PRO	PRE*PRO
Initial BW, g	256.56 ± 11.67	248.90 ± 10.53	251.44 ± 12.38	247.23 ± 9.91	0.68	0.08	0.31
Final BW, g	362.92 ± 16.68	350.17 ± 14.59	348.75 ± 12.71	342.08 ± 15.65	0.16	0.22	0.69
Gain, g	106.36 ± 9.90	101.28 ± 4.49	97.31 ± 8.85	94.86 ± 9.28	0.09	0.39	0.76
Feed intake, g/4-wks	624.75 ± 13.33^a^	614.32 ± 5.44^a^	554.31 ± 10.87^b^	560.94 ± 14.32^b^	0.01	0.75	0.16
FCR	5.91 ± 0.56	6.08 ± 0.25	5.74 ± 0.62	5.97 ± 0.71	0.62	0.50	0.91

*CELL (5% of cellulose), CELPRO (5% of cellulose + 1% of probiotics), OATS (5% of oatmeal), and OATPRO (5% of oatmeal + 1% of probiotics) groups

Prebiotics and probiotics are abbreviated as PRE and PRO, respectively

Probiotics contained *L. plantarum* (1 * 10^6^), *B. subtilis* (1 * 10^6^), and *S. cerevisiae* (1 * 10^6^)

Values indicate mean ± SD (n = 10)

^a,b^Mean within a same column with no common superscript differ significantly among the treatment groups (*p* < 0.05)

**Table 2:** Effect of prebiotics and probiotics on the relative organ weights (g/100 g BW)

Item	Treatment group*				Significance (*p* value)		
	CELL	CELPRO	OATS	OATPRO	PRE	PRO	PRE*PRO
Liver, g/100 BW	4.11 ± 0.56	3.80 ± 0.48	3.57 ± 0.36	3.89 ± 0.53	0.32	0.98	0.18
Spleen, g/100 BW	0.25 ± 0.03	0.24 ± 0.02	0.22 ± 0.06	0.24 ± 0.03	0.09	0.24	0.19
Intestinal mucosae, g/100 BW	0.57 ± 0.17	0.69 ± 0.19	0.58 ± 0.18	0.54 ± 0.13	0.40	0.58	0.31

*CELL (5% of cellulose), CELPRO (5% of cellulose + 1% of probiotics), OATS (5% of oatmeal), and OATPRO (5% of oatmeal + 1% of probiotics) groups

Prebiotics and probiotics are abbreviated as PRE and PRO, respectively

Probiotics contained *L. plantarum* (1 * 10^6^), *B. subtilis* (1 * 10^6^), and *S. cerevisiae* (1 * 10^6^)

Values indicate mean ± SD (n = 5)

### Blood biochemical profiles

The blood triglyceride level was significantly (*p* < 0.05) affected by dietary PRE, indicating that the rats fed an oatmeal diet resulted in lower triglyceride than those fed a cellulose diet (Table 3). The plasma AST level of rats fed a PRO supplemented diet was significantly (*p* < 0.05) lower than those fed a non-PRO supplemented diet. The biochemical components including albumin, total protein, cholesterol, glucose, ALT, BUN, and uric acid were unaffected by PRE type or PRO (Table 3).

**Table 3:** Effect of prebiotics and probiotics on the blood biochemical profiles

Item	Treatment group*				Significance (*p* value)		
	CELL	CELPRO	OATS	OATPRO	PRE	PRO	PRE + PRO
Albumin, g/dl	3.22 ± 0.13	3.18 ± 0.13	3.30 ± 0.08	3.26 ± 0.15	0.17	0.48	1.00
Total protein, g/dl	6.28 ± 0.22	6.40 ± 0.30	6.56 ± 0.21	6.24 ± 0.26	0.59	0.38	0.08
Triglyceride, mg/dl	95.00 ± 16.30^a^	94.80 ± 14.84^a^	52.75 ± 15.54^b^	64.20 ± 13.48^b^	0.01	0.44	0.43
Cholesterol, g/dl	72.20 ± 5.93	79.00 ± 10.41	75.60 ± 8.76	72.20 ± 3.11	0.58	0.58	0.11
Glucose, mg/dl	211.67 ± 47.43	193.50 ± 40.80	176.00 ± 33.05	166.33 ± 13.48	0.16	0.52	0.84
AST, U/L	120.75 ± 33.89	84.40 ± 9.53	110.25 ± 41.97	82.00 ± 11.45	0.62	0.03	0.76
ALT, U/L	69.60 ± 7.63	51.60 ± 9.13	63.20 ± 17.77	50.00 ± 15.57	0.87	0.09	0.23
Blood urea N, U/L	15.3 ± 2.40	14.46 ± 0.97	14.18 ± 1.78	12.53 ± 1.53	0.06	0.113	0.589
Uric acid, U/L	3.28 ± 1.88	2.66 ± 1.34	2.70 ± 1.17	2.58 ± 1.32	0.63	0.59	0.72

*CELL (5% of cellulose), CELPRO (5% of cellulose + 1% of probiotics), OATS (5% of oatmeal), and OATPRO (5% of oatmeal + 1% of probiotics) groups

Prebiotics and probiotics are abbreviated as PRE and PRO, respectively

Probiotics contained *L. plantarum* (1 * 10^6^), *B. subtilis* (1 * 10^6^), and *S. cerevisiae* (1 * 10^6^)

Values indicate mean ± SD (n = 5)

^a,b^Mean within a same column with no common superscript differ significantly among the treatment groups (*p* < 0.05)

### Small intestinal disaccharidase activity

**Fig. 1:**
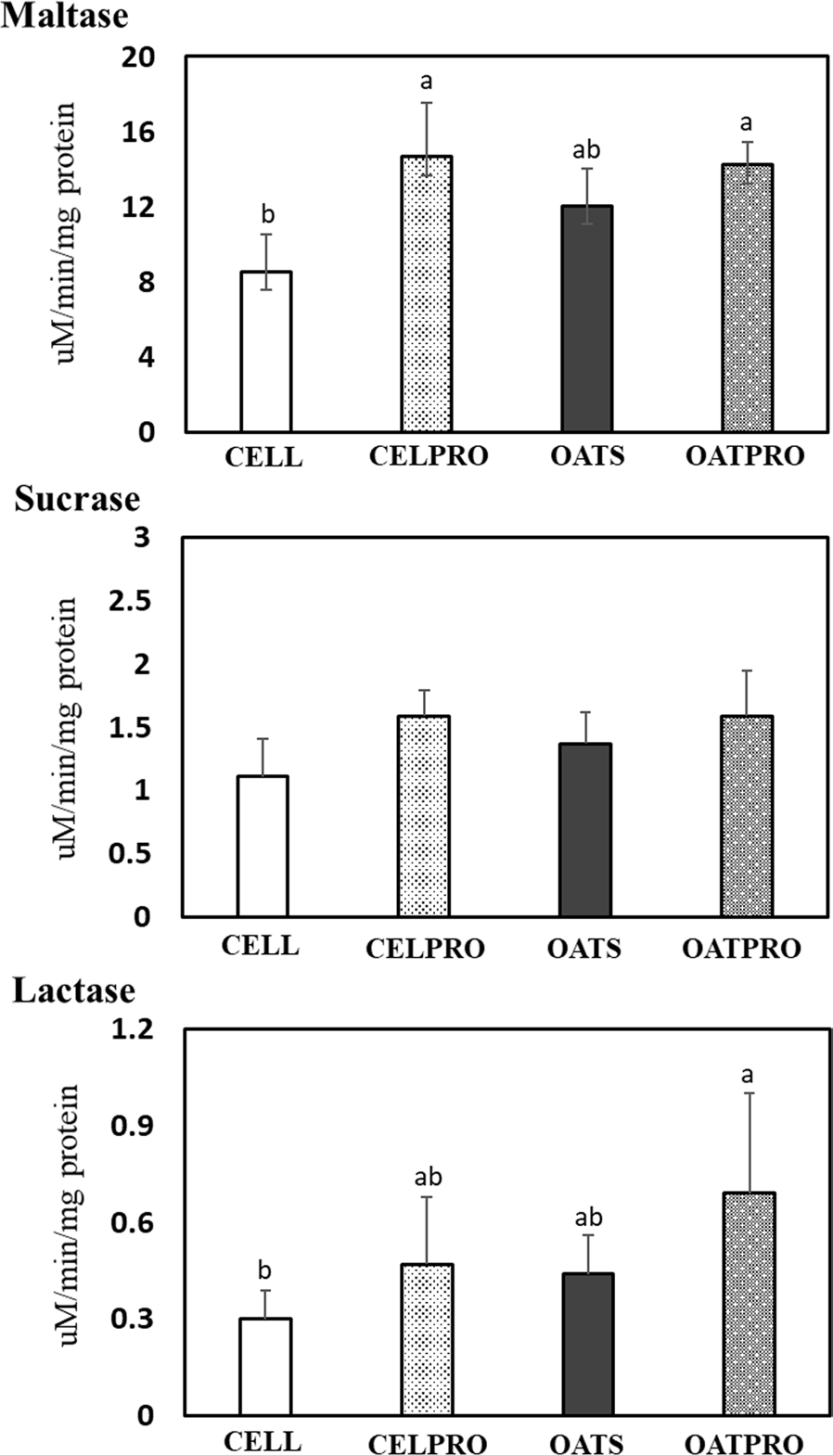
Effect of prebiotics and probiotics on the activity of disaccharidase in the small intestinal mucosa. *CELL (5% of cellulose), CELPRO (5% of cellulose + 1% of probiotics), OATS (5% of oatmeal), and OATPRO (5% of oatmeal + 1% of probiotics) groups. Values indicate mean ± SD (n = 5). ^a,b^Mean values in each panel with no common superscript differ significantly among the treatment groups (*p* < 0.05). The *p* values of maltase indicated as follows: PRE (*p* = 0.11), PRO (*p* = 0.01), and PRE*PRO (*p* = 0.07). The *p* values of sucrase indicated as follows: PRE (*p* = 0.32), PRO (*p* = 0.01), and PRE*PRO (*p* = 0.03). The *p* values of lactase indicated as follows: PRE (*p* = 0.07), PRO (*p* = 0.03), and PRE*PRO (*p* = 0.01)

The effect of dietary PRE type or PRO on the specific activity of intestinal disaccharidase is depicted in Fig. 1. Intestinal maltase, sucrase and lactase activities were significantly (*p* < 0.05) higher in rats fed the diet supplemented with PRO compared with rats fed the diet without PRO. However, the dietary PRE type did not affect intestinal hydrolase activity in rats. There was a significant (*p* < 0.05) positive interaction in the specific activities of sucrase and lactase, indicating that dietary PRE and PRO have a synergetic effect on intestinal disaccharidase activity.

### Microbial colony forming units (CFU)

**Fig. 2:**
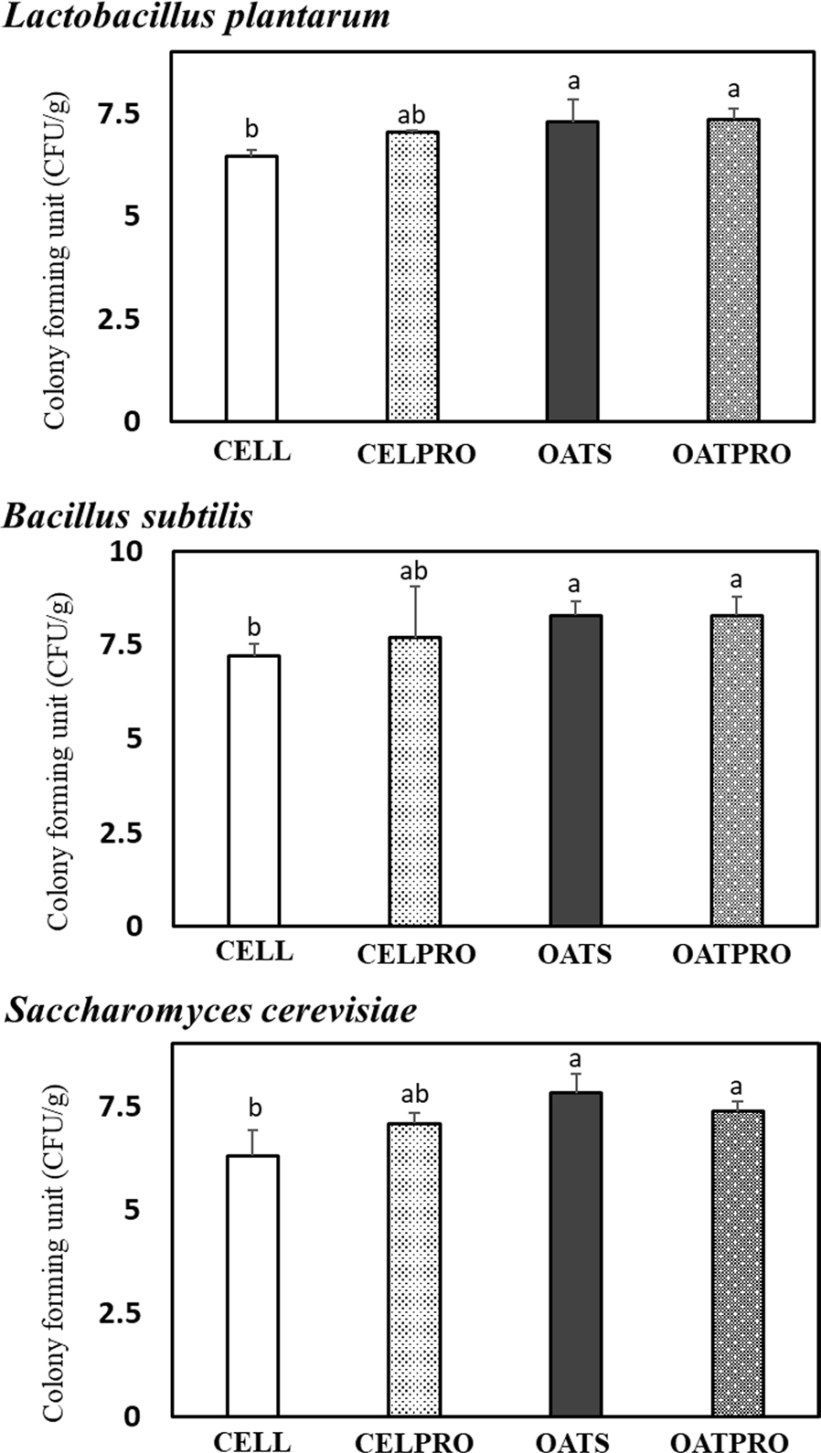
Effect of prebiotics and probiotics on the microbial counts (CFU/g) in the fecal contents. *CELL (5% of cellulose), CELPRO (5% of cellulose + 1% of probiotics), OATS (5% of oatmeal), and OATPRO (5% of oatmeal + 1% of probiotics) groups. Values indicate mean ± SD (n = 5). ^a,b^Mean values in each panel with no common superscript differ significantly among the treatment groups (*p* < 0.05). Prebiotics and probiotics are abbreviated as PRE and PRO, respectively. The *p* values of *L. plantarum* indicated as follows: PRE (*p* = 0.01), PRO (*p* = 0.09), and PRE*PRO (*p* = 0.17). The *p* values of *B. subtilis* indicated as follows: PRE (*p* = 0.01), PRO (*p* = 0.30), and PRE*PRO (*p* = 0.23). The *p* values of *S. cerevisiae* indicated as follows: PRE (*p* = 0.01), PRO (*p* = 0.53), and PRE*PRO (*p* = 0.04)

The effect of dietary PRE type or PRO on fecal microbial CFU is presented in Fig. 2. The CFU of *L. plantarum, B. subtilis,* and *S. cerevisiae* in fresh feces was significantly (*p* < 0.01) affected by the dietary PRE type. Rats fed an oatmeal supplemented diet showed a significant (*p* < 0.01) increase in the CFU of these microbes when compared to those fed a cellulose supplemented diet. However, there was no significant effect of PRO on the CFU of these microbes, although rats fed the diet fortified with PRO tended to increase (*p* < 0.09) in *L. plantarum* compared with those fed the non-PRO diet. There was a significant (*p* < 0.05) positive interaction in the CFU of *S. cerevisiae*, indicating that dietary PRE and PRO have a synergetic effect on the growth of yeast in the GI tract. Therefore, the type of PRE was the most important factor in establishing beneficial microbes of the GI tract from this study.

### Pro-inflammatory cytokines and *s*IgA

**Fig. 3:**
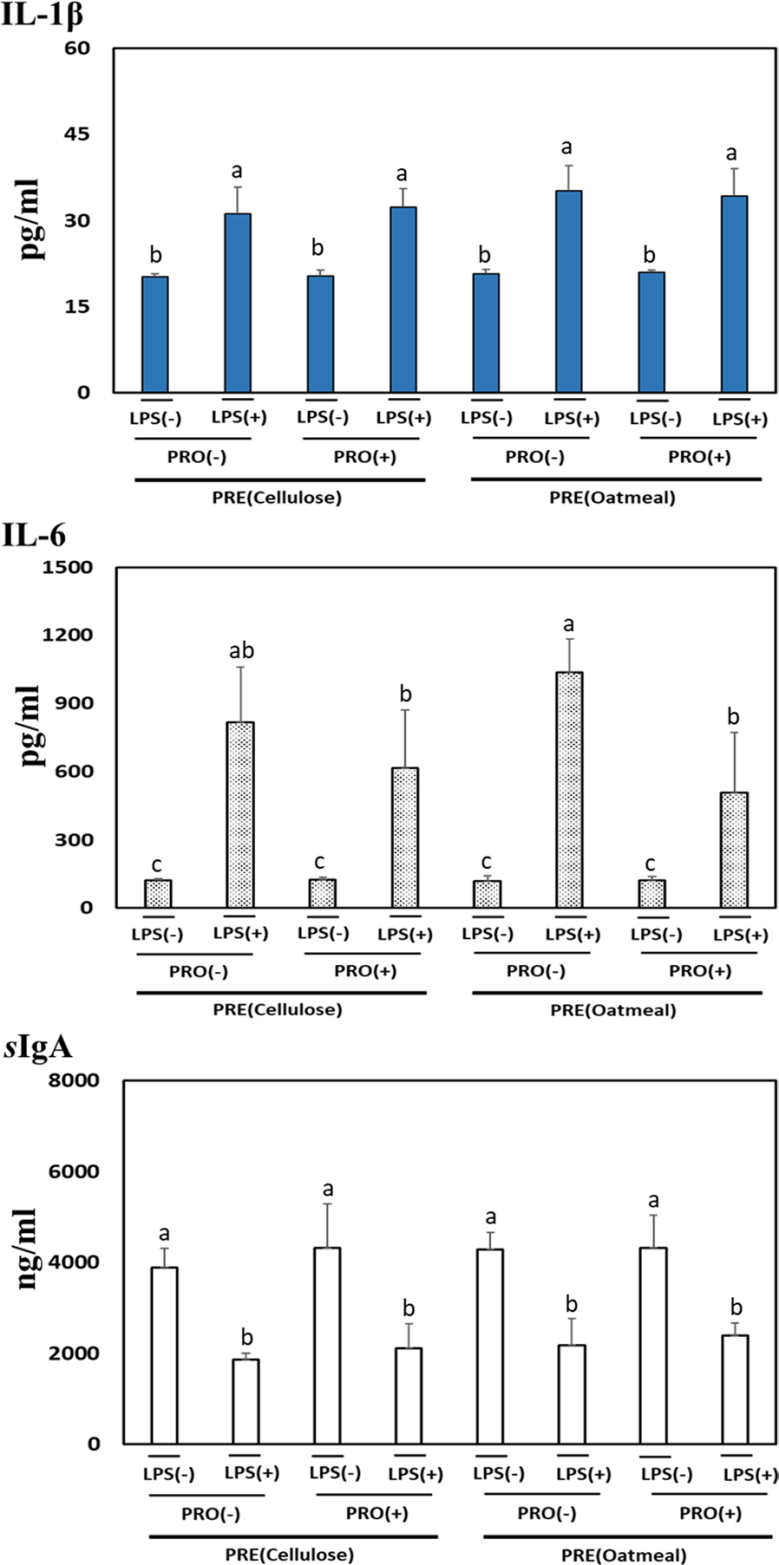
Effect of prebiotics and probiotics on IL-1β, IL-6, and *s*IgA levels in the blood. Values indicate mean ± SD (n = 5). ^a,b^Mean values in each panel with no common superscript differ significantly among the treatment groups (*p* < 0.05). Prebiotics and probiotics are abbreviated as PRE and PRO, respectively. The *p* values of IL-1β indicated as follows: PRE (*p* = 0.26), PRO (*p* = 0.79), LPS (*p* = 0.01), PRE*PRO (*p* = 0.48), PRE*LPS (*p* = 0.72), PRO*LPS (*p* = 0.73), and PRE*PRO*LPS (*p* = 0.48). The *p* values of IL-6 indicated as follows: PRE (*p* = 0.66), PRO (*p* = 0.01), LPS (*p* = 0.01), PRE*PRO (*p* = 0.05), PRE*LPS (*p* = 0.83), PRO*LPS (*p* = 0.01), and PRE*PRO*LPS (*p* = 0.09). The *p* values of *s*IgA indicated as follows: PRE (*p* = 0.26), PRO (*p* = 0.01), LPS (*p* = 0.01), PRE*PRO (*p* = 0.91), PRE*LPS (*p* = 0.34), PRO*LPS (*p* = 0.45), and PRE*PRO*LPS (*p* = 0.25)

**Fig. 4:**
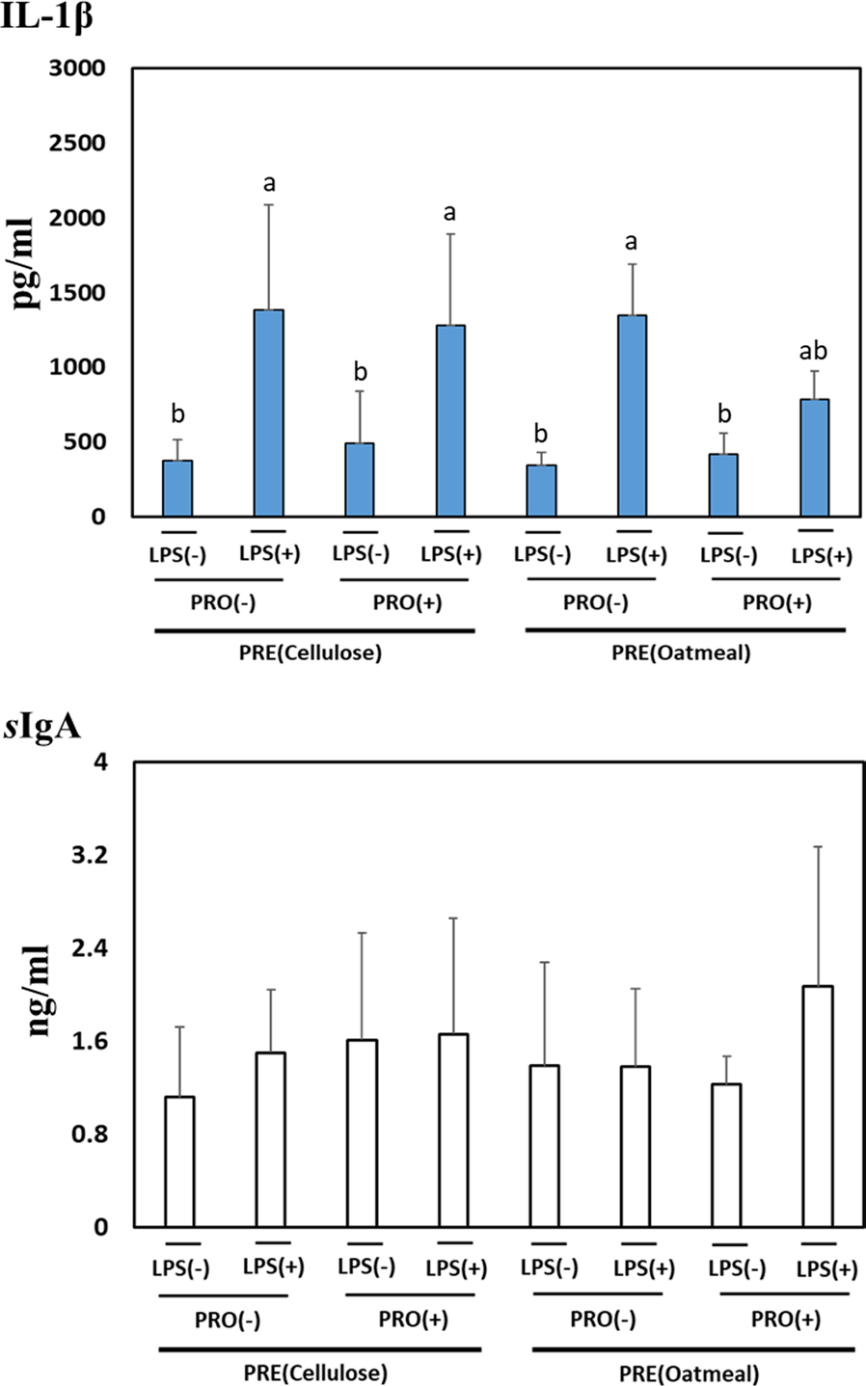
Effect of prebiotics and probiotics on IL-1β, and *s*IgA levels in the small intestinal mucosa. Values indicate mean ± SD (n = 5). ^a,b^Mean values in each panel with no common superscript differ significantly among the treatment groups (*p* < 0.05). Prebiotics and probiotics are abbreviated as PRE and PRO, respectively. The statistical *p* value of IL-1β indicated as follows: PRE (*p* = 0.26), PRO (*p* = 0.19), LPS (*p* = 0.01), PRE*PRO (*p* = 0.20), PRE*LPS (*p* = 0.36), PRO*LPS (*p* = 0.08), and PRE*PRO*LPS (*p* = 0.38). The statistical significance of *s*IgA indicated as follows: PRE (*p* = 0.40), PRO (*p* = 0.09), LPS (*p* = 0.09), PRE*PRO (*p* = 0.52), PRE*LPS (*p* = 0.38), PRO*LPS (*p* = 0.26), and PRE*PRO*LPS (*p* = 0.09)

Immediately after the 4-week feeding trial, each treatment group was divided into two sub-groups, which were injected with saline, or LPS (1 mg/kg BW) to induce inflammatory response in rats fed the PRE or PRO diet. The effects of dietary PRE type or PRO on the levels of IL-1β, IL-6, *s*IgA in blood and mucosal tissues are shown in Figs. 3 and 4, respectively. The level of blood *s*IgA in rats fed the PRO supplemented diet was significantly (*p* < 0.05) higher than those fed the non-PRO diet. The injection of LPS to rats significantly (*p* < 0.01) increased pro-inflammatory cytokines including IL-1β and IL-6 in the blood (Fig. 3). In particular, the LPS-treated rats fed the diet supplemented with PRO showed a significant (*p* < 0.05) decreased blood IL-6 level compared with those fed the diet with non-PRO, suggesting that dietary PRO alleviated the inflammatory response in LPS-treated rats. In the intestinal mucosal tissues, the injection of LPS also significantly (*p* < 0.05) affected the level of IL-1β (Fig. 4). The PRO supplemented diet did not significantly affect mucosal *s*IgA level compared with the non-PRO diet, although PRO diet tended to increase (*p* < 0.09) in mucosal *s*IgA level in rats. However, there was no significant effect of PRE type on pro-inflammatory cytokines and *s*IgA in the blood and mucosal tissues of LPS-treated rats.

## Discussion

The physiochemical properties of prebiotics in synbiotics may lead to wide variations in the composition of microbiota, digestive functions and immunity in the GI tract, although synbiotics have beneficial effects on the intestinal milieu of animals. Thus, we investigated the effects of PRE (insoluble vs. soluble type) and PRO (non-probiotics vs. probiotics) on the intestinal functions and immunological biomarkers in rats fed an AIN-93G diet. To control dietary PRE components in feed ingredients, the AIN-diet was provided to the rats in the study. We hypothesized dietary PRE in combination with PRO might affect growth performance, since cellulose or oatmeal as a type of PRE had a different digestible energy in rats. As a result, PRE and PRO did not affect body weight for the 4-week feeding trial but decreased ad libitum feed intake by 10% in rats fed an oatmeal-added diet. To address the reason, we can infer that higher digestible energy content in an oatmeal-added diet could be one of factors to decrease feed intake compared with those in a cellulose-added diet. Oatmeal contained approximately 35% of starch [16], which can be used for digestible energy source in rats. Another possible explanation for decreased feed intake in rats fed an oatmeal-supplemented diet is that high level of β-glucan in oatmeal, which is highly viscous fermentable fiber, might induce gastric distension and satiating effects in the host [17]. Our result is in agreement with the study of Tian et al. [18], who reported that fiber type in isocaloric diet significantly affected feed intake but not body weight in rats. Based upon other studies with AIN-93 diet with isocaloric synbiotic supplement, PRE and PRO supplements showed a limited effect on body weight [19–21]. By contrast, several epidemiological studies with human reported that dietary synbiotics decreased body weight [22, 23]. This result presumably appears to be due to a lower intake of caloric density or indigestible ingredients in prebiotics-rich synbiotics.

The PRE and PRO did not affect the weights of the liver, spleen, and small intestinal mucosa of rats in the study. The result is consistent with the result of a study, which reported that cellulose- or inulin based AIN-93 diet did not differ in the weights of the liver and spleen in mice [19]. Therefore, PRE and PRO would not affect the weights of organs such as the liver, spleen, and small intestine.

In blood biochemical indices, some positive effects of PRE and PRO supplements were observed. The blood triglyceride of rats fed an oatmeal added-diet was markedly lower than those fed a cellulose-added diet. It is speculated that triglyceride-lowering effect in rats fed an oatmeal-added diet was partly due to a significant decrease in feed intake. Consumption of high calories than the body burns can lead to a high blood triglyceride level in animals [24]. By contrast, the cholesterol-lowering effect of soluble fiber as reported by several studies [25–28] was not observed in this study. Presumably, blood cholesterol-lowering effect in rats fed an oatmeal-added diet was very limited because the amount of β-glucan present in the experimental diet was quite low. In addition, we found a significantly decreased AST level in rats fed the PRO-added diet compared with the non-PRO diet, indicating that PRO had positive effects on live functions [29].

Alteration in intestinal microbial composition appears to have a direct effect on intestinal digestive functions. In this study, intestinal maltase, sucrase and lactase activities significantly increased in rats fed the PRO supplemented diet. This result is similar to the previous study of Yang et al. [30], which reported that PRO containing *Lactobacilli* significantly increased intestinal lactase, sucrose, and maltase activity in rats. Collington et al. [31] also reported that the inclusion of PRO resulted in a significant increase in intestinal sucrase and lactase activities before weaning piglets. A study [32] also reported that GI microbiota affected intestinal functions, including digestive enzyme activity in the gut. Therefore, increased intestinal digestive enzyme activity in rats fed PRO was attributed to a healthy environment and efficient turnover rate of absorptive villi in the intestine.

In this study, the CFU of *L. plantarum, B. subtilis,* and *S. cerevisiae* in feces was remarkably affected by dietary PRE type. Rats fed an oatmeal diet significantly increased all three microbial CFU compared with those fed the cellulose diet. This observation is consistent with a study [33], which indicated that mice fed 10% fructans showed a greater abundance of fecal *Lactobacillus* than those fed 10% cellulose. Oatmeal supplement as soluble PRE selectively stimulated the growth of beneficial microbes including *L. plantarum,* and *B. subtilis*, since the different properties of PRE may lead to different composition of gut microbiota and fermented metabolites [15]. Although PRE such as cellulose, β-glucan, oligosaccharide, inulin, etc. is not digestible by digestive enzymes, each fiber was fermented into a different type of metabolites, which diversely affects the proliferation of enteric microbes in the GI tract. Thus, the reason why oatmeal supplement increased the CFU of fecal microbes in the study was that soluble fiber might provide more fermentable substrates for the proliferation of beneficial microbes in the GI tract compared with cellulose supplement.

As the settlement of beneficial microbiota plays a vital role in regulating gut immunity [7], we investigated the effects of insoluble or soluble PRE present in PRO on the level of pro-inflammatory cytokines (IL-1β and IL-6) and *s*IgA. More than any other cytokines, the IL-1β is primarily associated with innate immunity, which is strongly associated with damaging inflammation [34]. IL-6, which is produced at the site of inflammation, plays a crucial role in inflammatory response, and exerts stimulatory effects on the innate and acquired immunity [35]. In addition, *s*IgA plays a key role in mucosal immune defense system against pathogens and neutralizes their toxins to lower the expression of pro-inflammatory genes [36]. As acknowledged in LPS-induced inflammatory responses [37], LPS injection to rats significantly provoked pro-inflammatory cytokines including IL-1β and IL-6 in this study. The level of IL-6 in LPS-treated rats was significantly affected by dietary PRO not by PRE type, indicating that PRO ameliorated pro-inflammatory response. The level of *s*IgA in the blood and mucosal tissues of rats was significantly increased by PRO only. Dietary PRE did not affect pro-inflammatory cytokine and *s*IgA in LPS-treated rats. Similar to the previous studies, mice fed PRO showed higher serum IgA and lower TNF-α and IL-6, which implied that dietary PRO can increase immune functions [38, 39]. PRO, live microbes that exert benefits to intestinal barrier functions, has positive effects on the host immune system [6]. Several underlying mechanisms in which PRO can affect the immune system are via the settlement of beneficial microbes, the fortification of epithelial barrier function and the modulation of inflammatory genes in the GI tract [5]. In particular, *Lactobacilli, Bacilli,* and *S. cerevisiae* were known to down-regulate pro-inflammatory genes associated with inflammatory signaling pathways including toll like receptor 4 (TLR4) and nuclear factor (Nf)-kB [5].

At present, PRE including β-glucan has been widely known to protect against infection by pathogenic microorganisms. It is evident that PRE, a soluble fiber that selectively serves as a substrate for the proliferation of beneficial gut microbes, could indirectly affect immune response [20]. Most importantly, β-glucan has immunostimulatory effects on the activation of the mucosal immune system [40]. Hence, the potential combination of PRE with PRO would exert the immunomodulatory effect on inflammatory responses in the host, although the interactions of PRO strains and PRE properties are still unclear. Previous studies indicated that dietary synbiotics were capable of alleviating inflammation responses in the GI tract and improving host health [20, 41]. A study with synbiotics consisting of oligosaccharide and *Lactobacilli* showed a significant reduction of NF-kB and TNF-α [13]. In accordance with this study, the intestinal mucosal *s*IgA level was significantly higher in response to dietary PRO in combination with PRE including insulin and oligosaccharide as PRE sources [20, 42].

## Conclusions

The present study demonstrated that oatmeal as a PRE source showed beneficial effects on the fecal microbial population compared with cellulose. In addition, dietary PRO positively affected intestinal digestive enzymes and immune functions, including increasing *s*IgA, and alleviating IL-6 level in the LPS-treated SD rats. Further studies are still necessary to understand how the synergistic interplay between PRO strains and PRE type is beneficially working as synbiotics in the host.

## Methods

### Experimental animals and design

The animal experiment was approved by a university institutional animal care and use committee (IACUC, 201901) and performed in compliance with committee guidelines. Male Sprague Dawley (SD, 5-week old) rats purchased from Samtako Co. (Osan, Korea) were kept in an environmentally controlled room (23 ± 2 °C) with a 12 h light/dark cycle after an acclimation of 1-week. Forty rats having a similar body weight were randomly allocated to 4 groups in a 2 × 2 factorial design (n = 10): cellulose (CELL, cellulose 5%), cellulose + probiotics (CELPRO, cellulose 5% plus 1% PRO), oatmeal (OATS, oatmeal 5%), and oatmeal + probiotics (OATPRO, oatmeal 5% plus 1% PRO) groups. After allocation, all rats were fed American Institute of Nutrition 93G (AIN-93G) diet for a 2-week of preliminary period to adjust purified diet. The PRO complex (D&A Co., Haman, Korea) containing *L. plantarum, B. subtilis,* and *S. cerevisiae* (> 10^6^ CFU) was added to PRE matrix including cellulose or oatmeal. Dietary cellulose as insoluble fiber and oatmeal as soluble fiber were purchased from Sigma-Aldrich (Saint Louis, MO, USA) and Quaker oats Co. (Chicago, IL, USA), respectively. All rats were given ad libitum to the AIN-93G purified diet for 4-week as shown in Table 4. Weekly body weights, daily food intakes, and feed conversion ratio (FCR) was calculated during the 4-week trial period. Immediately after the 4-week feeding trial, each group was divided into two sub-groups (n = 5), which were injected intraperitoneally to saline or lipopolysaccharide (LPS, Cat No. L2630, Sigma-Aldrich, Saint Louis, MO, USA) to induce inflammatory response in rats.

**Table 4:** Composition of AIN-93G based purified diets used for the experiment

Items	Treatment group*			
	CELL	CELPRO	OATS	OATPRO
Ingredients (%)				
Casein	20.00	20.00	20.00	20.00
Soybean oil	7.00	7.00	7.00	7.00
Sucrose	10.00	10.00	10.00	10.00
Dextrose	13.20	13.20	13.20	13.20
Corn starch	38.80	38.80	38.80	38.80
Cellulose^1^	5.00	5.00	–	–
Oatmeal^2^	–	–	5.00	5.00
Cysteine	0.30	0.30	0.30	0.30
TBHQ	0.0014	0.0014	0.0014	0.0014
Choline	0.20	0.20	0.20	0.20
AIN 93-Mineral mix	3.50	3.50	3.50	3.50
AIN 93-Vitamin mix	1.00	1.00	1.00	1.00
H_2_O	1.00	–	1.00	–
Probiotics^3^	–	1.00	–	1.00
Total	100	100	100	

*CELL (5% of cellulose), CELPRO (5% of cellulose + 1% of probiotics), OATS (5% of oatmeal), and OATPRO (5% of oatmeal + 1% of probiotics) groups

^1^Cellulose was supplemented as a source of insoluble prebiotics

^2^Oatmeale was supplemented as a source of soluble prebiotics

^3^Probiotics containing *L. plantarum* (1 * 10^6^), *B. subtilis* (1 * 10^6^), and *S. cerevisiae* (1 * 10^6^) were premixed with 5% of cellulose or 5% of oatmeal

### Tissue harvesting and preparation

At 12 h after the injection of saline or LPS, all animals were sacrificed with anesthetizing ether following 6 h deprival of diet. After opening the abdominal cavity, blood collected from hepatic inferior vena cava was put in a tube coated with sodium heparin and plasma was isolated by the centrifugation with 3000 × *g* (Vision, VS-15,000 CF, Hanil Sci. Co., Korea). After bleeding, the liver, spleen, and small intestine were collected, weighed and rapidly frozen into liquid nitrogen. On the day of tissue preparation, the small intestine was perfused with 0.9% ice-cold saline and gently squeezed to remove the digesta. The whole intestine was cut, weighed, and rinsed three times with ice-cold saline. Mucosal tissue was harvested by gently scraping the internal surface of the intestinal segment with a glass slide on an ice-cold aluminum pan. After that, residual fat and digesta in the harvested mucosa were removed by suspending the tissue in an equal volume of the saline buffer followed by centrifugation at 10,000 × *g* at 4 °C for 12 min. All tissues were stored at − 70 °C until further assay.

### Blood biochemical profile analyses

Blood biochemical components including aspartate aminotransferase (AST), alanine aminotransferase (ALT), blood urea nitrogen (BUN), glucose, triglyceride, cholesterol, and uric acid were assayed using a clinical biochemical analyzer (Mindray, BS-120, Mindry Bio Medical Electronics co., Shenzhen, China).

### Determination of specific activities of disaccharidase

The harvested mucosal tissues were homogenized with fivefold of the mannitol buffer, and aliquots of the resulting homogenate were stored at − 70 °C until used. The homogenate aliquot, upon thawing, was diluted with an equal volume of 2% triton X-100 to release brush-border enzymes from the membrane fraction. The activity of disaccharidase including maltase, sucrose, and lactase was determined by a procedure modified from that of Dahlgvist’s method [43]; the end-product (glucose) was determined using an ELISA with its wavelength set at 450 nm. The specific activity of each enzyme corresponded to a yield of the end-product per minute by one mg of protein, respectively. Protein content of the mucosal homogenate was assayed by the bicinchronic acid (BCA) method using a protein assay kit (Pierce, Rockford, IL, USA).

### Determination of immunoassay for IL-1β, IL6, and sIgA

Blood and intestinal mucosal extraction were used for the quantification of inflammatory cytokines and secretory immunoglobulin A (*s*IgA). The mucosal tissues homogenized with fivefold of saline were centrifuged at 3000 × *g* for 10 min to collect tissue extraction for further assay. In vitro ELISA assay kits including rat interleukin (IL)-1β (Cat. No. RLB00, R&D Systems, USA) and IL-6 (Cat. No. R6000B, R&D Systems, USA) were used for the quantitative measurement of these cytokines in blood and mucosal extraction according to the protocol booklet from the supplier. After following appropriate procedures, the relative amount of rat IL-1β or IL-6 was quantified by measuring the absorbance at 450 nm with ELISA. Rat *s*IgA ELISA kit (Cat. No. CSB-E08412r, CUSABIO, China) was applied to examine the concentration of *s*IgA with reference to the manual of production. After following the assay manual procedures, the amount of *s*IgA was quantified by measuring the absorbance at 450 nm with ELISA.

### Microbial colony forming units (CFU*)*

The ten-fold serial dilution method using sterilized H_2_O was performed to determine the number of colony forming units (CFU) in fecal contents (1 g). The CFU of *L. plantarum* was determined using MRS agar (Difco, USA) after incubation in an aerobic chamber at 37 °C for 48 h. The CFUs of *B. subtilis* and *S. cerevisiae* were enumerated on Tryptic Soy agar (Difco, USA) and YPD agar (Difco, USA), respectively after aerobic incubation at 37 °C for 24 h.

### Statistical analysis

All values are expressed as means ± standard deviation (SD). Statistical analyses were performed two-way (PRE and PRO) or three-way (PRE, PRO, and LPS) analysis using Proc GLM (general linear model) procedure [44]. A *p* value of < 0.05 was considered statistically significant effect of treatment (PRE, PRO, LPS or interaction of prebiotics, probiotics and LPS). When a significant effect (*p* < 0.05) of factor was observed, the Tukey multiple range test was applied to compare the means among treatment groups.

## Data Availability

The datasets used and/or analyzed during the current study are available from the corresponding author on reasonable request.

## Abbreviations

AIN: American Institute of Nutrition
ALT: Alanine aminotransferase
AST: Aspartate aminotransferase
B: *Bacillus*
BCA: Bicinchronic acid
BUN: Blood urea nitrogen
CFU: Colony forming units
GI: Gastrointestinal
GLM: General linear model
IACUC: Institutional animal care and use committee
IL: Interleukin
L: *Lactobacillus*
LPS: Lipopolysaccharide
Nf: Nuclear factor
PRE: Prebiotics
PRO: Probiotics
*s*IgA: Secretory immunoglobulin A
S: *Saccharomyces*
SD: Sprague Dawley
SD: Standard deviation
TLR4: Toll like receptor 4
TNF-α: Tumor necrosis factor-α
